# Introduction to the Special Issue “Scientific Breakthroughs to Fruit and Vegetable By-Product Valorization in the Food Sector”

**DOI:** 10.3390/foods12142726

**Published:** 2023-07-17

**Authors:** Amalia Conte, Matteo A. Del Nobile

**Affiliations:** Department of Agricultural Sciences, Food and Environment, University of Foggia, Via Napoli 25, 71122 Foggia, Italy

We are pleased to present this Special Issue, which includes five papers that highlight important research activities in the field of fruit and vegetable by-product valorization. The goal of this Special Issue is to broaden current knowledge about the possibility of recycling fruit and vegetable by-products for sustainable innovations in the food sector. The food industry annually produces tons of by-products during processing. Globally, researchers estimate that, in the food supply chain, the percentages account for about 40–50% of the total material are discarded. Apart from aiming to reduce losses and waste at all levels of the food chain, it is also possible to recycle by-products rather than throwing them away. This could limit both the economic and environmental impacts. Fruit and vegetable by-products are the most abundant waste because they can occur during pre- and post-harvesting processes, preparation, and processing. Industrial fruit and vegetable by-products are very different from one another because of the differences in raw materials and industrial processes. They occur as leaves, peels, seeds, pulps, and mixtures of these. It is worth noting that food and vegetable by-products are all rich in valuable bioactive compounds, such as simple sugars, carbohydrates, polysaccharides, pectin, and fibers. They also have bioactive molecules such as phytochemicals, antioxidants, antimicrobials, phenolics, flavonoids, and/or carotenoids. Over time, thanks to the growing awareness of the great potential of their active compounds, they have been employed in several industrial fields for cosmetic and pharmaceutical purposes. Due to valuable bioactive molecules, food by-products have huge potential for being recycled within the food sector, as they are very useful to human nutrition and promote many healthy functions [1,2]. In the food sector, fruit and vegetable by-products can be used in production line as raw materials to obtain new functional products with high health benefits. They can be used as natural preservatives to extend food products’ shelf life, or can be included in polymeric systems to develop active and/or intelligent packaging solutions or to create bio-nanocomposite films. With this perspective in mind, the current Special Issue brings together five papers that cover the following topics: (i) by-product characterization; (ii) by-products for food shelf life extension; (iii) by-products to develop intelligent packaging systems; and (iv) by-products to develop nanocomposite bio-based systems.

The publication by Cairone et al. [3] deals with the chemical characterization of *Punica granatum* L. seed oil. The seeds were subjected to a classic Soxhlet extraction with n-hexane or extraction with supercritical CO_2_, assisted by ethanol, and the oil quality was strongly influenced by the adopted extraction procedure. The resulting oils were evaluated using different techniques to highlight differences in the triacylglycerols composition. Results showed a prevalence of up to 75% of punicic acid in the triacylglycerol mixture, with a clear preponderance in the extract conducted using supercritical fluids. Punicic acid plays a role in immune function, with effects on eicosanoid production and the suppression of inflammatory responses, the prevention of cardiovascular disease, and in delaying the onset and progression of obesity. In addition, the antiradical potential demonstrated through DPPH analysis showed that the extract obtained with supercritical CO_2_ was much more active.

Two articles were published on by-products recycled for shelf-life extension. The first [4] aimed to investigate the possibility of using the whole pomegranate (juice, peel, and seeds), according to the zero-waste approach, to prolong the shelf-life of fresh fish. Zero-waste manufacturing involves designing products and processes which produce no waste; all parts of the food, including the by-products, are completely utilized in the food formulation [5]. In the study by Panza et al. [4], a preliminary antimicrobial in vitro test was carried out with peel and seeds as ground and re-ground powders. Then, the entire fruit, in the right proportions of juice and relative by-products, was added to fresh fish burgers and stored at 4 °C for 1 month. The results from the in vitro test clearly indicated that the peel was abundantly more effective than seeds and that the ground peel powder had a slightly higher antimicrobial activity than the same re-ground powder. The results from the shelf-life test showed that all fortified burgers were more appreciated from both microbiological and sensory points of view compared to the control. It was clearly noted that the samples with pomegranate remained acceptable for about 2 or 3 weeks, depending on the type of powder added (ground or re-ground), while the control sample became unacceptable within a few days, due to microbial proliferation. This study could represent a valuable example of the zero-waste approach. However, to explore this opportunity in depth, a proper comparison between energy costs and emissions linked to the zero-waste approach and those associated with traditional by-product disposal is highly recommended. The second case-study of by-products used for food preservation was research by Panza et al. [6] dealing with ready-to-eat prickly pears. In particular, peels from prickly pears were dehydrated, ground, and reused in an alginate-based solution to be applied as a coating to fresh-cut prickly pears. Prickly pear peels were abundantly reviewed in the literature [7,8], and their high bioactive compounds, dietary fiber, fatty acids, lipid classes, sterols, fat-soluble vitamins, and b-carotene contents are well recognized. Due to these well-known properties, the peels of prickly pears were recycled to preserve the fruit quality. To that aim, a shelf-life test was carried out with coated and uncoated fresh-cut fruits, properly packaged in bags, to monitor weight loss, microbial spoilage, pH, and sensory properties under refrigerated storage conditions. A simple coating without any peel addition was also considered. Important differences were recorded between the coated and uncoated fruit. When compared to the uncoated fruit, coated prickly pears presented lower weight loss and delayed microbial and fungal proliferation. Comparing the simple coating and the coating containing peels, a better preservation of fresh-cut prickly pears was recorded using the coating and by-products. Specifically, the sensory quality was better retained when peels were used, due to the good preserving properties of this powder, which contributed to a perfect maintenance of color, odor, and texture for the entire observation period.

Another interesting application of by-products was found in the study of Otálora González et al. [9], where anthocyanins and betalains from agro-industrial by-products were properly valorized. Specifically, the authors developed intelligent packaging using microparticles generated from *Brassica oleracea* and *Beta vulgaris* L., which contained anthocyanins or betalains. Different authors reported that films containing natural pigments can be used as intelligent packaging to monitor the freshness of food products. The above authors included a mix of microparticles in the formulation of edible films based on cassava starch, for the first time. The films with active compounds were suitable for sensing the deterioration of packaged and chilled hake. The labels containing the natural pigments anthocyanins/betalains showed high sensitivity to total volatile basic nitrogen content. In addition, the color change of the label was completely consistent with fish muscle deterioration. The authors also observed that the presence of microparticles as fillers increased the mechanical strength and the hydrophobicity of the films.

Another case-study published in the Special Issue is the research of Bigi et al. [10], dealing with waste orange peels as a source of cellulose nanocrystals (CNCs) to develop nanocomposite films. A current trend in research consists of reinforcing the biopolymer matrix with nanomaterials to overcome the technical criticisms of packaging. In this context, CNCs have been widely studied as reinforcing agents for different biopolymers due to their renewability, biocompatibility, and low cost. In the last few years, many efforts have been dedicated to isolating valuable CNCs from agricultural by-products such as pineapple, banana, orange, tomato peels, grape pomace, and sugarcane bagasse. The inclusion of CNCs increased the films’ tensile strength, light barrier, and water vapor barrier properties while reducing their water solubility. Orange peels represent one of the most promising sources of CNCs due to their high cellulosic content and their extended availability. The paper of Bigi et al. [10] provides an optimized protocol for the extraction of cellulose from discarded orange peels and its conversion to CNCs. The films were strengthened by the addition of extracted CNCs as fillers. These bio-nanocomposites were also enriched with a synthetic cationic surfactant characterized by a broad biocidal activity, biodegradability, nontoxicity, and prospective applicability as an additive.

Taken together, these studies are clear evidence of how the recycling of by-products can be valorized for food and packaging applications. The innovative and exciting research included in this Special Issue highlights the interest in and potential of this emerging area, addressing some of the most pressing global issues. The development of this topic and the exploration of the potential use of food by-products remain very active research areas. Given the context, the aim of subsequent research would be to focus simultaneous attention on the main interdisciplinary factors that could make industrial food by-products an effective entry point to mitigate the great food waste problem. Therefore, it is necessary to combine the technical feasibility assessments with an analysis of the real economic and environmental impact.

We sincerely hope that readers will find this Special Issue informative and interesting.
